# Factors Associated with Larval Control Practices in a Dengue Outbreak Prone Area

**DOI:** 10.1155/2014/459173

**Published:** 2014-09-17

**Authors:** Mariam Mohamad, Mohamad Ikhsan Selamat, Zaliha Ismail

**Affiliations:** Faculty of Medicine, Universiti Teknologi MARA, Jln Hospital, Sungai Buloh Campus, 47000 Sungai Buloh, Selangor, Malaysia

## Abstract

In order to reduce the risk of dengue outbreak recurrence in a dengue outbreak prone area, the members of the community need to sustain certain behavior to prevent mosquito from breeding. Our study aims to identify the factors associated with larval control practices in this particular community. A cross-sectional study involves 322 respondents living in a dengue outbreak prone area who were interviewed using a pretested questionnaire. The level of knowledge about *Aedes* mosquitoes, dengue transmission, its symptoms, and personal preventive measures ranges from fair to good. The level of attitude towards preventive measures was high. However, reported level of personal larval control practices was low (33.2%). Our multiple logistic regression analysis showed that only those with a good level of attitude towards personal preventive measure and frequent attendance to health campaigns were significantly associated with the good larval control practices. We conclude that, in a dengue outbreak prone area, having a good attitude towards preventive measures and frequent participation in health campaigns are important factors to sustain practices on larval control.

## 1. Introduction

Almost 50% of the world's population is living in dengue infection risk areas, a mosquitoes-borne fever with 50–100 million cases reported annually and about 2.5% affected died [1].* Aedes* mosquitoes particularly* Ae. aegypti* and* Ae. albopictus* are two known vectors that play the crucial role of transmitting dengue virus from an infected host (mainly human) to another human being. To date, the main measures for vector control include destroying the mosquitoes' larval breeding sites either by chemical, biological, or environmental management and space spraying using insecticides to kill the adult mosquitoes [2]. Both of these measures need collaboration from the community as well as the public health authorities. Thus, evidences to support local community participation to destroy the mosquitoes are essential.

Malaysia is a highly endemic country for dengue infection, which experienced 3- to 5-year cycles of outbreaks [3]. In 2013, the incidences of dengue and dengue haemorrhagic fever were 143.3 and 2.6 per 100,000 populations, respectively, with mortality rate of 0.31 per 100,000 [4]. The control program is highly subsidized by the government and the bulk of the prevention and control measures are done by the public health authority [5]. Localities are monitored by number of dengue cases reported and are considered as having outbreak when there is more than one case of dengue occurring within 14 days from the date of onset of the first case. These areas would then undergo intensive measures (standard operating procedure) for dengue outbreak control. Apart from adulticiding by space spray and using insecticide, antidengue campaigns which include community participation in the activities of mass cleaning of the area, health education activities, for example, talks, exhibition, and distribution of health education materials regarding dengue infection and control measures are also carried out. As for larval control practices, the residents are educated on how to identify and destroy breeding areas and the use of larvacides (chemical or biological). These practices required the people living in the community particularly those living in the highly endemic or outbreak prone area to practice and sustain the behavior in order to reduce the incidence of dengue infection that could be fatal to them.

Our study aims to find out the level and factors that are associated with the practice of larval control among the residents who live in a dengue outbreak prone area. We hope that we would be able to identify the sustenance factor for the behavior in this particular group of high risk community.

## 2. Material and Methods

A community based cross-sectional study was carried out in an apartment type living area in Selangor, Malaysia. The area was a known dengue outbreak prone area, defined as an area which recently (within a year) had a dengue outbreak episode. The area had been subjected to many dengue control activities in accordance with the national guideline which includes antidengue campaign before our study was done.

We calculated the estimated sample size of 320 respondents based on 95% confidence level, 5% precision with prevalence proportion (level of good larval control practices) of 25%, and a nonresponse rate of 10% using the single proportion formula [6, 7].

The study area consists of 10 blocks of 6-storey low cost apartment, where each floor consists of 10 units of living quarters, giving a total of 600 living quarters. The occupancy rate was approximately 60% which gives rise to 360 occupied living quarters. We conducted cluster sampling using occupied living quarters as the cluster and the head of all occupied living quarters as our respondents. If the head of the household was not available after the second visit, the oldest person in the house was selected to be the respondent.

Respondents were interviewed using pretested questionnaire by trained interviewers. Verbal consent was gathered before the interview after the objective of the study was informed to the respondent. The questionnaire contained 4 sections.  Section 1 covers the sociodemographic details and Section 2 contained questions on knowledge regarding* Aedes* mosquitoes identification and behavior (7 questions), dengue transmission (9 questions), dengue symptoms (20 questions), and prevention measures (7 questions).  Section 3 was on attitude towards larval control practices (8 questions) while Section 4 was on self-reported frequency of participation in antidengue campaign held in the area and frequency of carrying out larval control practice (4 questions) using 5-point frequency scale. There were four questions related to larval control practices, that is, the frequency of eliminating containers with stagnant water surrounding their home, frequency of covering water containers at home, frequency of cleaning water containers at home, and lastly frequency of putting ABATE (or larvicides) in a water filled container in their home.

Data were entered and analyzed using IBM SPSS Statistics 20. After descriptive analysis, the frequency of larval control practices was then categorized into two groups, that is, poor practice (those with a total score of 15 and below) and good practice (score of 16 and above) and was treated as the dependent variable in our inferential analysis. Simple logistic regression analysis was done to determine the important independent variables for larval control practice; the variables with *P* value of <0.25 were selected to be included in the multiple logistic regressions. Those with *P* value of <0.05 were considered as statistically significant. Multicolinearity, interaction, and model fit analyses were also done to the model.

Our study was approved by the Research and Ethics Committee of Universiti Teknologi MARA.

## 3. Results

Out of 360 living quarters identified, 322 respondents from each living quarter were interviewed (89.4%). The nonresponse was either because there were no people in the house after two visits or people refused to participate.

The mean age of our respondents was 33.9 years (SD 11.0) with a range between 15 and 65 years old and mean monthly household income of RM 2252 (SD 1258). More than half (54.3%) were females and majority attained secondary and tertiary education (88.2%) and are married (72.4%). About 10.6% of the respondents had been diagnosed as having dengue infection before. About 52.8% of our respondents perceived themselves as having high risk for dengue infection (Table 1).

The mean score for knowledge regarding* Aedes* mosquitoes was 5.69 ± 1.28 over 7.00, knowledge about dengue transmission was 4.59 ± 1.93 over 8.00, knowledge about dengue symptoms was 12.03 ± 4.00 over 20, and knowledge of personal preventive measures was 5.52 ± 1.49 over 7.00 (Table 2). All the means of the four knowledge scopes were between fair and good level.

As for attitude towards personal preventive measures, the mean score was considerably good, that is, 32.50 ± 5.87 over 40.

About 75% of our respondents never use bed nets and 40% never use mosquito coils, while nearly 15% never use mosquitoes aerosol. As for the larval control practice, those who reported never destroying or eliminating containers with stagnant water inside, surrounding their home were 7.5%, never covering water container at home were 11.5%, never cleaning the water container at home were 8.1%, and, lastly, never using larvicide (ABATE) in a water filled container in their home were 36.6%. Using these four questions, we later categorized them into two groups, that is, poor practice (those with a total score of 15 and below) and good practice (score of 16 and above).

Our simple logistic regression analysis shows that being unemployed and housewife and having more knowledge on* Aedes* mosquitoes, on dengue symptoms, and on personal preventive measures together with a better attitude towards personal preventive measures, always or most of the time joining dengue campaign, were the significant associated factors for good larval control practices (Table 3).

In our multiple logistic regression analysis, it was found that having better attitude towards personal preventive measures (*P* = 0.004; adjusted OR 1.08 (1.02, 1.14)) and always or most of the time joining campaigns (adjusted OR 3.83 (1.96, 7.50)) were significant (Table 4).

## 4. Discussion

Our study found that in a dengue outbreak prone area, the level of knowledge among the residents on* Aedes* mosquitoes, dengue transmission, its symptoms, and personal preventive measures ranges from fair to good. Even attitude towards preventive measures was high. However, the ultimate target to have a frequent level of personal practices of larval control was low (33.2%). Although there is no “standard” level to measure adequacy or acceptable level for this particular behavior or practices, we consider 33% as low. A study in periurban areas in Laos reported larval breeding control practices in the range of 11 to 50% for 6 practices under study (covering water containers, cleaning water containers regularly, treating water in water containers, not storing water, cutting down vegetation around the home, and disposing of old tyres) [8]. One recent qualitative study had identified the barriers to sustained self-prevention which include lack of self-efficacy, lack of perceived benefit, low perceived susceptibility, and unsure perceived susceptibility [9] while others had reported the barriers as misconceptions about dengue from outdated educational materials, “invisibility” of dengue compared with chronic diseases, and lack of acceptance of responsibility for dengue prevention [10]. We suggest to further study the community living in dengue outbreak areas on the barriers for the low frequency of reported larval control practices.

Interestingly, we found only two factors that are independently associated with good larval control practice which are good attitude towards preventive measures (adjusted OR 1.08, 95% CI 1.03, 1.14) and frequent self-reporting of joining antidengue campaigns held in the community (adjusted OR 3.83, 95% CI 1.96, 7.50). Other sociodemographic factors such as age, sex, educational level, and household income were not significantly associated with larval control practices in this particular community, although some studies have shown the associations of these factors with the practices [11, 12]. The possible explanation was the almost homogenous sociodemographic characteristics in our studied community which result in the insignificance associations.

Studies correlating knowledge and practices on dengue prevention have shown inconsistent results. In our study, the levels of knowledge regarding* Aedes* mosquitoes, dengue transmission, symptoms, and the prevention practices were not significantly associated with the frequency of practices of larval control. Although many studies and theoretically good practice are related to the good knowledge level [11, 13, 14], some studies did show that good knowledge level does not usually correlate with practice [15–19]. We concur with Phuanukoonnon et al. [20] that, during an outbreak, information received through health education activities can raise awareness of dengue infection but is probably insufficient to sustain larval control practices. They identified several barriers which include insufficient control agents and incompatibility of control practices with people's beliefs.

More than one-third (37.0%) of our respondents never attend antidengue health campaign held in the community. The proportion is slightly less than one study (43.3%) in Penang regarding never attending any health promotion campaign [21]. We did not study the barriers for those not attending. However, we found that one very important factor is that in order for the community living in a dengue outbreak prone area to change behavior to a high satisfactory level of larval control practices, they need to participate in antidengue campaign held in the neighborhood. This also stressed on the importance of such campaign. Unfortunately, the implementation of antidengue campaign does pose many challenges such as its cost benefits and community mobilization. Therefore, public health authorities should consider the local community priorities in designing antidengue campaign in their area to encourage more participation. Ways to attract people to attend the antidengue campaign should be plotted and applied especially to the high risk area for dengue outbreak.

We identify the limitations of our results that have been obtained from a cross-sectional design where the outcome (larval control practices) and the independent factors were studied at one point of time and thus the temporal effect could be challenged. However, we are very careful in using the term as associations and not risk factors or predisposing factors. We also subjected our data to multiple logistic regression analysis in order to reduce the effect of confounders in our result. Currently, there are no standardized (or validated) questions to assess knowledge, attitude, and practices (KAP) for* Aedes* control and dengue infection, and this is evidenced from many inconsistencies in the findings of relating practices with the knowledge and attitude as discussed above. We tried to reduce this limitation by pilot-testing the questions earlier and also checked with a content expert before conducting the study.

We recommend extending this study to involve both dengue and nondengue outbreak prone areas and subjecting the communities to intervention packages. It would include the development of educational activities with specific messages with the intention to increase good attitudes and ensure behavior change regarding larval control practices. To measure the outcome more objectively, entomologist should be involved in the study to quantitate vector density.

## 5. Conclusion

Larval control practices are among the required behaviors in order to control dengue infection, especially in dengue risk areas. Our study showed that having a good attitude towards dengue prevention measures and frequent participation in antidengue campaigns are associated with frequent larval control practices in this particular area.

## Figures and Tables

**Table 1:** Sociodemographic characteristics of the respondent (*n* = 322).

	Frequency	%	Mean	SD
Mean age (years)			33.9	11.0
Mean household income per month (RM) (*n* = 257)			2252	1258
Mean number of people in household			4.8	1.9
Sex				
Male	147	45.7		
Female	175	54.3		
Highest education level				
No formal and primary	38	11.8		
Secondary and tertiary education	284	88.2		
Current employment status				
Employed	161	50.0		
Unemployed and housewife	120	37.3		
Student	36	11.2		
Pensioner	5	1.6		
Marital status				
Married	233	72.4		
Single/divorced/separated	89	27.6		
Citizenship				
Malaysian	309	96.0		
Non-Malaysian	13	4.0		
Ethnic group				
Malay	257	79.8		
Non-Malay	65	20.2		
Risk perception				
Strongly disagree	28	8.7		
Disagree	61	18.9		
Neutral	63	19.6		
Agree	120	37.3		
Strongly agree	50	15.5		
Ever diagnosed as having dengue infection (*n* = 321)				
Yes	34	10.6		
No/Do not know	287	89.1		

Grand total	**322**			

**Table 2:** Mean knowledge score and percentage of respondents reported personal adult mosquitoes control and larval control practices.

	Frequency	%	Mean	SD
Knowledge on *Aedes* mosquitoes (score 0 to 7)			5.69	1.28
Knowledge on dengue transmission (score 0 to 8)			4.59	1.93
Knowledge of dengue symptoms (score 0 to 20)			12.03	4.00
Knowledge on personal preventive measures (score 0 to 7)			5.52	1.49
Attitude towards personal preventive measures (score 8 to 40)			32.50	5.87
Use of mosquitoes aerosol				
Never	47	14.6		
Few or sometimes	154	47.8		
Most of the time and always	121	37.6		
Use mosquitoes coils				
Never	128	39.8		
Few or sometimes	119	37.0		
Most of the time and always	75	23.3		
Use bed nets				
Never	240	74.5		
Few or sometimes	52	16.1		
Most of the time and always	30	9.3		
Join the campaign				
Never	119	37.0		
Few or sometimes	142	44.1		
Most of the time and always	61	18.9		
Level of practice of larval control				
Poor (score of 15 and below)	215	66.8		
Good (score of 16 and above)	107	33.2		

**Table 3:** Factors associated with level of larval control practice from simple logistic regression analyses.

Factors	Crude OR 95% C.I.	Wald	df	Sig.
Age (years)	1.01 (0.99, 1.03)	1.29	1	0.26
Monthly household income (RM)	1.00 (1.00, 1.00)	0.33	1	0.56
Number of people living in a household	1.03 (0.91, 1.17)	0.23	1	0.63
Sex				
Female	REF			
Male	1.24 (0.78, 1.99)	0.83	1	0.36
Highest education level				
No formal and primary	REF			
Secondary and tertiary education	0.74 (0.37, 1.47)	0.75	1	0.39
Employment status				
Employed	REF			
Unemployed and housewife	1.73 (1.05, 2.84)	4.58	1	0.03
Student	0.962 (0.43, 2.15)	0.01	1	0.92
Pensioner	1.67 (0.27, 10.30)	0.30	1	0.58
Citizenship				
Malaysian	REF			
Non-Malaysian	1.77 (0.58, 5.39)	0.99	1	0.32
Ethnic group				
Non-Malay	REF			
Malay	0.97 (0.54, 1.72)	0.01	1	0.91
Marital status				
Still married	REF			
Single/divorced/separated	0.72 (0.42, 1.23)	1.46	1	0.23
Knowledge on *Aedes* mosquitoes	1.27 (1.04, 1.55)	5.33	1	0.02
Knowledge on dengue transmission	1.04 (0.92, 1.17)	0.37	1	0.55
Knowledge of dengue symptoms	1.06 (1.00, 1.13)	4.01	1	0.04
Knowledge on personal preventive measures	1.18 (1.00, 1.40)	3.93	1	0.05
Attitude towards personal preventive measures	1.08 (1.03, 1.14)	9.59	1	0.002
Risk perception				
Low risk	REF			
Neutral	0.87 (0.44, 1.73)	0.16	1	0.69
Moderate and high risk	0.92 (0.54, 1.58)	0.09	1	0.76
Ever diagnosed as having dengue infection				
No	REF			
Yes	1.23 (0.56, 2.67)	0.26	1	0.61
Join the campaign				
Never	REF			
Few and sometimes	1.51 (0.87, 2.62)	2.13	1	0.15
Most of the time and always	4.09 (2.12, 7.91)	17.55	1	0.00

**Table 4:** Factors associated with level of larval control practice in a community in a dengue outbreak prone area.

	Crude OR^a^ (95% CI)	Adjusted OR^b^ (95% CI)	Wald statistics	df	*P* value
Attitude towards personal preventive measures	1.08 (1.03, 1.14)	1.08 (1.02, 1.14)	8.24	1	0.004
Join the campaign					
Never	1.00	1.00	16.24	2	0.000
Few and sometimes	1.51 (0.87, 2.62)	1.39 (0.79, 2.43)	1.32	1	0.25
Most of the time and always	4.09 (2.12, 7.91)	3.83 (1.96, 7.50)	15.43	1	0.000

^a^Simple logistic regression, ^b^multiple logistic regression.

The model reasonably fits well. Model assumptions are met. There are no interaction and multicolinearity problems.
